# The Current Status, Bioactivity, Food, and Pharmaceutical Approaches of *Calocybe indica*: A Review

**DOI:** 10.3390/antiox11061145

**Published:** 2022-06-10

**Authors:** Meghna Shashikant, Aarti Bains, Prince Chawla, Melinda Fogarasi, Szabolcs Fogarasi

**Affiliations:** 1 Department of Food Technology and Nutrition, Lovely Professional University, Phagwara 144411, Punjab, India; meghna2899@gmail.com; 2 Department of Biotechnology, CT Institute of Pharmaceutical Sciences, South Campus, Jalandhar 144020, Punjab, India; aarti05888@gmail.com; 3 Department of Food Engineering, University of Agricultural Sciences and Veterinary Medicine of Cluj Napoca, Calea Mănăstur 3–5, RO-400372 Cluj-Napoca, Romania; melinda.fogarasi@usamvcluj.ro; 4 Department of Chemical Engineering, Faculty of Chemistry and Chemical Engineering, Babeş-Bolyai University, 11 Arany Janos Street, RO-400028 Cluj-Napoca, Romania; 5 Interdisciplinary Research Institute on Bio-Nano-Sciences, Babeş-Bolyai University, 42 Treboniu Laurian Street, RO-400271 Cluj-Napoca, Romania

**Keywords:** *Calocybe indica*, therapeutic application, bioactivity, mushroom, antioxidants

## Abstract

Over the past few years, mushrooms have been extensively explored in the field of pharmaceutical and food science, and researchers are heading toward the search for vital components with a higher safety margin and multitarget applications. Moreover, among all age group populations, mushroom consumption has increased immensely owing to their great nutritional aspects, desirable organoleptic properties, and aroma. In addition, mushrooms continue to generate much attention chiefly in their consumption as food, as a cure for different ailments, as well as a vital commodity globally, owing to their dietary, antioxidant, and therapeutic values. Mushrooms are considered one of the important and suitable diets for patients having multiple types of diseases. Additionally, due to potential immunomodulatory effects, quality protein, and low fat, and cholesterol content, mushrooms are used as an important ingredient for food formulation. Therefore, this review article provides detailed information on *Calocybe indica* as they are the third most important commercially grown mushroom following button and oyster mushrooms. This review brings tangible evidence that milky white mushrooms are a great source of natural components and antioxidants with potential application in pharmaceuticals and in treating and managing different diseases. Several food applications of milky white mushrooms have also been discussed and reviewed.

## 1. Introduction

Mushrooms are epigeous macrofungi with an umbrella-shaped structure where spores are produced, consisting of the following two phases of growth: the reproductive (fruiting bodies) and the vegetative phase (mycelia) [1]. Globally, there are over 14,000 different species of mushrooms; however, among these, approximately 2000 species are edible mushrooms, and as a consequence, about 200 species of mushrooms have been commercially produced for therapeutic formulations and human consumption [2,3]. Additionally, edible mushrooms have attained high demand as a staple food source and are highly acceptable for their potential textural, flavor, medicinal, and tonic properties. Chemically, these are composed of vital phytochemicals (phenols, flavonoids, terpenes, terpenoids, steroids), and desirable nutritious compositions that are high in protein, fiber, minerals (phosphorus, iron potassium, magnesium, barium, aluminum, manganese, copper, zinc, boron, nickel, and chromium) and vitamins (B1, B2, B12, C, D, and E) [4,5]. The nutritional, pharmaceutical, bioremediation, and biodegradation qualities of mushrooms are expanding by the day, and have accelerated in recent years [6]. Apart from this, mushrooms have over 100 medicinal functions and their key medicinal uses include antibacterial, antifungal, antiparasitic, antiviral, anticancer, antioxidant, antidiabetic, antiallergic, anticholesterolemic, immunomodulating effects, cardiovascular, hepatoprotective effects, and detoxification [7].

The milky mushroom has a wide range of sizes, with numerous stems growing from a single base. From base to cap, the mushroom is completely white, does not fade with age or handling, does not bruise or discolor, and has a robust meaty stem and a firm disc-like top. The caps resemble button mushrooms and can be dome-shaped to almost convex in form as they mature, whereas pale gills protrude from beneath the cap. Milky white mushrooms have a soft yet delightfully chewy texture and flavors comparable to button mushrooms [8]. The advantages of milky mushrooms over other strains are the convenient method of cultivation, low investment, appealing fruiting body, desirable milky white color, extended shelf life, nutritious value, and a shorter growth period [4]. The production of milky mushrooms majorly depends on the quality of spawn and substrate, and, according to several researchers, the best substrate for the cultivation of milky mushrooms was paddy straw [5]. Due to their biochemical composition and antioxidant properties, they have been reported to prevent oxidative damage by free radical and reactive oxygen species (ROS) and may prevent the onset of carcinogenesis, physical injury, infection, aging, and cardiovascular diseases. Hence, *Calocybe indica* is considered a better proxy for *Pleurotus ostreatus* notably due to their longer shelf life in tropical regions [9].

To maintain health, the application of mushrooms has been used in several industries, including the food industry, to form functional, nutraceutical, and healthy foods, in pharmacology for the development of ayurvedic medicines, antibiotics, and in other fields of research. Furthermore, mushrooms have long been used as a dietary supplement somewhere in the middle between the greatest veggies and the best animal protein sources in diverse cultures, and they are cultivated and consumed for their palatable taste [10]. It has been documented that *Calocybe indica* is a rich source of vitamins, minerals, proteins, and amino acids, and since they are low in fat content, they make an ideal diet for heart patients [11]. Additionally, they are a good source of bioactive polysaccharides, such as β-glucans and polyphenols (flavonoids, alkaloids, and triterpenoids). Therefore, these active compounds may be responsible for scavenging processes, enhancing antioxidant activity [12], anti-diabetic, anti-cancer [13], and anti-lipid peroxidation characteristics. Pleuran, lentinan, schizophyllan, β-glucans, mannans, chitin, hemicellulose, galactans, and xylans polysaccharides are regarded as probiotic properties of *Calocybe indica* polysaccharides [14]. Individuals are discovering healthier functional food alternatives that are rich in metabolic goodness and provide protection against disease, and mushrooms perfectly fit into the group of functional foods [15]. Several scientific investigations have highlighted different pharmaceutical properties of mushrooms such as antioxidant, antimicrobial, anticancer, and immunomodulatory activity on various extracts of milky mushroom [16]. Few proteins target immune cells known as fungal immunomodulatory proteins (FIPs) and form a new of group bioactive proteins [17]. Mushrooms are improving in the field of complementary and alternative medicine (CAM) as a functional food due to their ability to modulate humoral and innate immunity as well as revitalize the weakened immune system. From *Calocybe indica*, proteins and glucans were isolated and both of these compounds showed immunostimulatory effects and also stimulated the activity of natural killer cells which kill cancerous cells directly [15]. These remarkable functional, physiochemical, and techno-functional properties of *Calocybe indica* mushroom make them a promising ingredient in the food and pharmaceutical industry. In consideration of the above circumstances, the present review aims to sum up the findings related to the status, cultivation, nutritional, bioactivity, food, and pharmaceutical prospects of *Calocybe indica* with schematic diagrams of the mechanism.

## 2. Current Status of *Calocybe indica*

### 2.1. Origin, Morphological and Physiological Features

*Calocybe indica* grows in the tropical climates of Africa, China, Malaysia, Singapore, Indonesia, and India, largely owing to its longer shelf life and adaptability to warm and humid conditions [17,18]. The name *Calocybe indica* was derived from the Ancient Greek terms kalos “pretty”, and cubos “head” and belongs to the following taxonomic group: Phylum: Basidiomycota, Class: Agaricomycetes, Order: Agaricales, Family: Tricholomataceae [19,20]. After button and oyster mushrooms, it has become the third most commercially cultivated mushroom in India [21].

**Figure 1:**
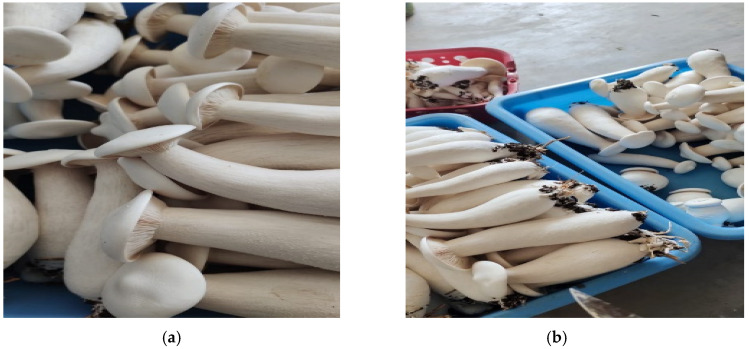
(**a**,**b**) The fruiting body of *Calocybe indica* collected from a farm in Chennai, Tamilnadu, India.

The cap of *Calocybe indica* averages from 10 to 14 cm in diameter and is white, umbrella-shaped, or convex in appearance (Figure 1a,b), while further flattening occurs as the mushroom ages while the stipe is bulbous and both the ring and the volva are absent [22]. They possess a distinctive farinaceous odor [23]. According to various studies, the pileus and gills are richer in protein (40–60%), lipid (30–60%), and ash content (5–10%) than the stipe, whereas the stipe is richer in fiber (40–50%) and carbohydrate (10–15%) [24].

For the increased productivity and nutrition of cultivated mushrooms, the optimal culture media, temperature, pH, and substrate must be identified and optimized accordingly [25]. *Calocybe indica* was classified as a thermo tolerant due to their ability to be cultivated in a warm climate ranging from 30 °C to 38 °C with a humid condition of 80% to 85% and hold a longer shelf life without the need for refrigeration [18]. A drastic negative effect on the mycelial growth of *Calocybe indica* was observed when the pH was less than 4.0 and maximum growth was reported at pH 6.0 [26]. Furthermore, distinct physiological changes occur in the life phases of mushrooms, such as changes in color, size, and form [27].

### 2.2. Cultivation

The milky mushroom is one of the finest edible mushrooms that can be cultivated throughout the year in the tropical climate of India. Furthermore, some of the characteristics that make it a better choice for mushroom producers and consumers are a simple cultivating process, minimal capital investment, and long shelf life [10]. Milky white mushroom farming is a labor-intensive and energy-intensive cultivation process involving the following six steps: spawn production, substrate pre-treatment, mushroom bed preparation, cropping room maintenance during spawn run, mushroom production, packing and spent mushroom substrate management. Mushroom cultivation generally occurs on a variety of cellulosic substrates and the most common lignocellulosic substrates used for mushroom cultivation include paddy straw, wheat straw, soybean straw, and sugarcane bagasse, cotton waste, and coconut coir pith. [5]. Spawning is carried out at a rate of 4% of the wet weight of straw containing 60–65% moisture. The casing is a crucial step in the development of spawn after it has finished its run. The pinhead initiation and the final yield are determined by the quality and quantity of the easing material used [28]. Furthermore, they are less prone to contamination and discoloration when subjected to controlled conditions. Additionally, the cost of the production of these species is inexpensive, suggesting that industrial production could involve a short crop cycle of approximately 7–8 weeks [18].

### 2.3. Casing

During the transition from the vegetative to reproductive phase, the casing layer plays a crucial function in initiating fructification. [29]. Casing materials must have a high water-holding capacity, physical support, moisture, and a good air space ratio to provide a gaseous exchange, porosity, and bulk density such as peat moss, loam soil, spent mushroom substrate, coconut coir, biogas slurry, farmyard manure, and so on [25]. Appropriate proportions of garden loam soil (50%), sand (25%), and farmyard manure (25%) were used to make the casing material. The light should be available for a substantial amount of time, and the resulting changes in the environment result in the commencement of fruiting bodies in the form of needles within 3–5 days, which mature in approximately a week. Mushrooms with a diameter of 7–8 cm are collected, twisted, cleaned, and packaged for selling in perforated polythene/polypropylene bags. For prolonged storage, mushrooms can be wrapped with cling film [5].

## 3. The Nutritional Profile of *Calocybe indica*

### 3.1. Proteins

Food and Agriculture Organization (FAO) provide that mushroom has contributed significantly to protein nutrition as a food item in developing countries such as India, which particularly depend on cereal-based diets. The interest in proteins from plant sources as an alternative to animal proteins has been growing in recent decades mainly due to their reduced production cost, abundant supply, and content of bioactive and phytochemical substances which can be fulfilled by mushroom proteins comprising high plant protein that is readily digested of which content in terms of dry weight can range from 10 to 40% [30,31]. The essential amino acid reports of mushrooms reveal that the proteins are deficient in sulfur-containing amino acids, including methionine and cysteine, but comparatively rich in threonine and valine. In a study by Chelladurai et al. (2021) [4], proteins of *Calocybe indica* were regarded as the dominant compound (14.11%) and recorded 20.2% protein from the caps of milky white mushrooms on a dry weight basis. Similarly, Subbiah et al. (2015) [18] reported 32.2% protein on a dry weight basis in a medium-sized milky mushroom. It was also found that the crude protein content of *Calocybe indica* is slightly lower than for other mushrooms considering that it has only 2.09 g/100 g while in *Agaricus bisporus* and *Pleurotus ostreatus* the protein content is 4.83 ± 0.04 and 3.22 ± 0.17, respectively, showing that it has a comparable nutritional composition to other important mushrooms [32,33].

### 3.2. Vitamins

Several mushrooms have been investigated for their vitamin content, and the results suggest that they are abundant in vitamins A, B-complex, C, D, and E. In a vitamin study conducted by Sumathy et al. (2015) [34] *Calocybe indica* was shown to be a strong source of vitamin B, followed by Vitamin E, A, and C, among the four vitamins measured. The findings of the analysis are consistent with the existing research of Sathish (2017) and Barros (2007) [35,36]. *Calocybe indica* might be an effective alternative to the vitamin diet because vitamins are vital in the diet of human beings and standard sources of vitamins are limited. According to a study conducted by Subbiah et al. (2015) [18], most mushrooms are high in vitamins and minerals, especially B complex vitamins (thiamine, riboflavin, pyridoxine, pantothenic acid, nicotinic acid, nicotinamide, folic acid, and cobalamin), as well as ergosterol and biotin. In this study, the vitamin A content in fresh and dry milky white mushrooms has been reported to be 0.35 mg and 0.275 mg per g, respectively. Vitamin C (a free radical scavenger and a well-known antioxidant and inhibitor of lipid peroxidation (LPO)) (1.03 mg/100 g) [37], vitamin E (tocopherol), (2.8 mg/g), and glutathione (0.025 nmole/g) were also found to be abundant in *Calocybe indica*.

### 3.3. Minerals

Mineral elements are also crucial for human health since they have physiological impacts on many organs and cellular functions [38]. According to research, *Calocybe indica*, similarly to other mushrooms, has a mineral mix, and its fruiting bodies contain a high degree of assimilable mineral elements [34]. In their analysis of the mineral content in dried mushrooms, Chelladurai et al. (2021) [4] demonstrated a good source of minerals. The maximum macro mineral present in *Calocybe indica* was potassium, followed by magnesium, phosphorus, and barium. Micro minerals such as iron, aluminum, manganese, copper, zinc, boron, nickel, and chromium were recorded. Similarly, according to a study of the nutritional analysis of mushrooms conducted by Alam et al., 2008 [24], the mineral contents of dried *Calocybe indica* were expressed in mg/100 g. Calcium, iron, zinc, magnesium, manganese, selenium, and arsenic were found to be present.

### 3.4. Carbohydrates

Polysaccharides are the most well-known and effective anti-tumor and immunomodulating compounds obtained from mushrooms. Numerous data on mushroom polysaccharides have been collected from hundreds of different species of β-glucans and are well known for their biological activity which is primarily related to the immune system; as a result, the greatest technique for preventing cancer cell proliferation appears to be activating and reinforcing the host immune system [39]. Mushrooms polysaccharides, namely the calocyban from *Calocybe indica*, have been developed and exploited as functional food substances, that proved to be and are considered outstanding representatives of D-glucans with common (1→3) or (1→6) β-linked glucose backbones and are distinguished by different patterns and degrees of branches [40]. Collectively, the most common monosaccharides-detected mushroom polysaccharides were glucose, galactose, fructose, xylose, mannose, fucose, rhamnose, arabinose, trehalose, and mannitol [7]. Numerous studies have inspected and revealed the potential abilities of mushroom polysaccharides in terms of such biological activities The heteropolysaccharide of *Calocybe indica* showed benefits in the antioxidant and anti-aging activities, raised the activities of SOD, CAT, GPx, levels of GSH, and lowered the levels of MDA in mice brain and serum [41]. They also provide neuroprotective activity against D-galactose-induced cognitive dysfunction, oxidative damage, and mitochondrial dysfunction in mice [42]. According to a study conducted by Alam et al. (2008) [24], *Calocybe indica* showed the highest carbohydrate content both in the fresh (6.8 ± 0.5 g/100 g) and dried (48.5 ± 2.4 g/100 g) form in comparison with *Pleurotus ostreatus*, *Pleurotus sajor caju*, *Pleurotus florida*.

### 3.5. Fatty Acids

In a study conducted by Chelladurai et al. (2021) [4], a gas chromatography equipped with a flame identification detector detected a total of 17 fatty acids in *Calocybe indica* among which stearic acid, lignoceric acid, myristic acid, lauric acid, palmitic acid, heneicosylic acid, pentadecyclic acid, margaric acid, and arachidic acid were saturated fatty acids and linoleic acid, elaidic acid, myristoleic acid, eicosapentaenoic acid, erucic acid, palmitoleic acid, gondoic acid, and dichomo-linolenic acid were unsaturated fatty acids. Linoleic acid and elaidic acid were the most abundant fatty acids in *Calocybe indica*.

Table 1 describes the different suitable methods for the estimation of the nutritional profile of *Calocybe indica*.

## 4. Bioactive Functions

**Figure 2:**
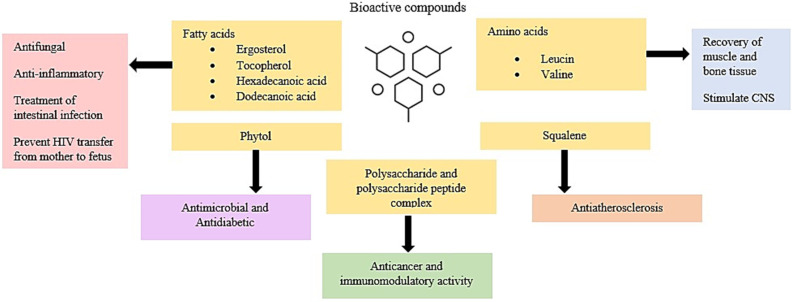
Bioactive compounds in milky mushrooms and their applications. CNS: central nervous system.

Mushroom species can be used as a natural source of bioactive components that have a distinct impact on human health and disease prevention, exhibiting pharmacological effects such as antidiabetic, antitumor, immunomodulating, cardiovascular, antimicrobial, hepatoprotective, and antioxidative effects [46]. Several extraction techniques have been used to prepare the mushroom extract including the hot aqueous extract of fresh fruit bodies [47], methanolic extract [41], ethanolic extraction, and modified solvent evaporation extraction [48], and hot water extraction [49]. Bioactive compounds found in edible mushrooms include phytochemicals (alkaloids, phenolic acids, flavonoids, carotenoids), fiber, polysaccharides, selenium, vitamins (e.g., niacin, thiamin, riboflavin, ascorbic acid, and vitamins B and D), and the significant antioxidants ergothioneine and glutathione, which may play a role in the prevention of cancer [50]. Figure 2 shows the bioactive compounds present in *Calocybe indica* including phytol, squalene, fatty acids, amino acids, polysaccharides, and protein-polysaccharide complexes. This species includes antioxidants and inhibits cancer cells [51,52], as well as prevents metastasis [53], UV radiation-induced inflammation [54], proliferation, apoptosis, and migration [53]. Researchers recently identified and documented the chemical composition of several substances with biological activity and secondary metabolites from *Calocybe indica* [55]. Several experiments demonstrated antioxidant, antidiabetic, anticarcinogenic, hepatoprotective, antimicrobial, antiproliferative, and hypertensive activities in *Calocybe indica* [11,56]. In a study by Mishra et al. (2014) [52], natural foods containing antioxidants were used to reduce oxidative damage, herein the methanolic extract of caps of Calocybe indica at a concentration of 1 mg/mL showed a total antioxidant activity of 45.31 ± 2.16 µM, whereas the stipes exhibited 25.78 ± 1.22 µM antioxidant activity at the same concentration proving that the caps of *Calocybe indica* have more antioxidant potential than that of stipes. According to several findings, Ghosh (2022) [15] concluded that the toxicological screening found a complete absence of anatoxin and phallotoxin. Herein, experimental rats feeding on mushroom water extract did not produce any toxic effect, whereas this could be consumed as a safe healthy food, indicating its potential to be developed as a non-toxic antimicrobial agent.

The applications of the bioactivity identified by several extraction methods are listed in Table 2.

### 4.1. Phenolic and Flavonoid Compounds

**Figure 3:**
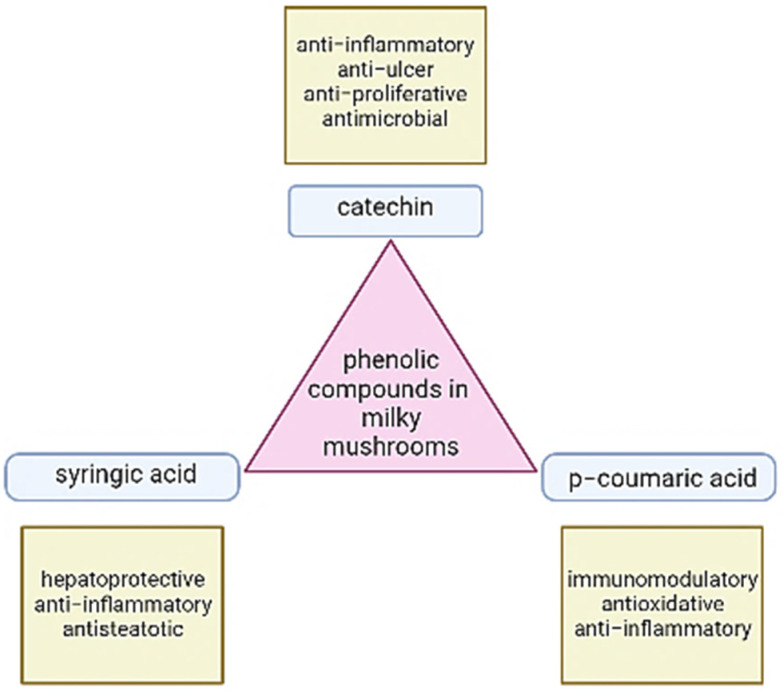
Phenolic compounds in the milky mushroom.

The presence of phenolic substances such as phenolic acids, hydroxycinnamic acids, lignans, tannins, flavonoids, hydroxybenzoic acids, stilbenes, and oxidized polyphenols has been linked to anti-inflammatory activities in the various mushrooms [60]. These substances have been characterized as free radical inhibitors, peroxide decomposers, metal inactivators, and oxygen scavengers [17]. Mushroom extracts have a high concentration of phenolic compounds that are mainly composed of one or more aromatic rings including one or more hydroxyl groups and can serve as hydrogen donors or electron donors, and possess metal ion-chelating characteristics [52]. Phenolic compounds present in milky mushrooms include catechin, syringic acid, p coumaric acid, and caffeic acid and their applications are listed in Figure 3. According to a study by Prameela (2020) [61], there is no specific association between the number of carbohydrates and pileus size, but there is a notable increase in the number of phenols as the size of the pileus grows.

### 4.2. Antimicrobial Activity

**Figure 4:**
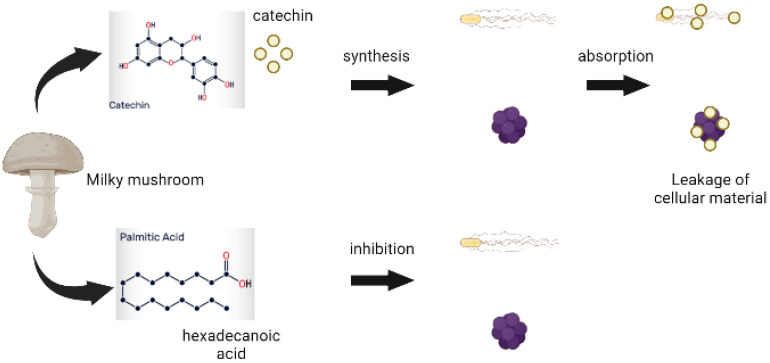
Antimicrobial activity of *Calocybe indica*.

Resistance to antibiotics by pathogenic microorganisms has been a global problem in recent years, and both Gram-positive and Gram-negative bacteria have diverse mechanisms against antimicrobial drugs, demanding the development of a novel and efficient alternative against these microorganisms [48]. Mushrooms contain a variety of bioactive chemicals with strong antimicrobial activity against both Gram-positive and Gram-negative bacteria. Many of the extracellular secretions of the mushroom mycelium have been proven to inhibit bacteria and viruses. Through this mechanism, the binding and synthesis of catechin and hexadecenoic acid to the bacterial cell wall are responsible for antimicrobial activity against *Escherichia coli* and *Staphylococcus aureus* (Figure 4). Furthermore, using the gas chromatograph-mass spectrometer (GCMS) approach, many phytocompounds such as ethyl tridecanoate, undecanoic acid ethyl ester, diallyl divinylsilane, and 3-phenyl-pyrrolo (2,3-) pyrazine were identified and reported to have antimicrobial characteristics [59].

### 4.3. Anti-Inflammatory Characteristics

**Figure 5:**
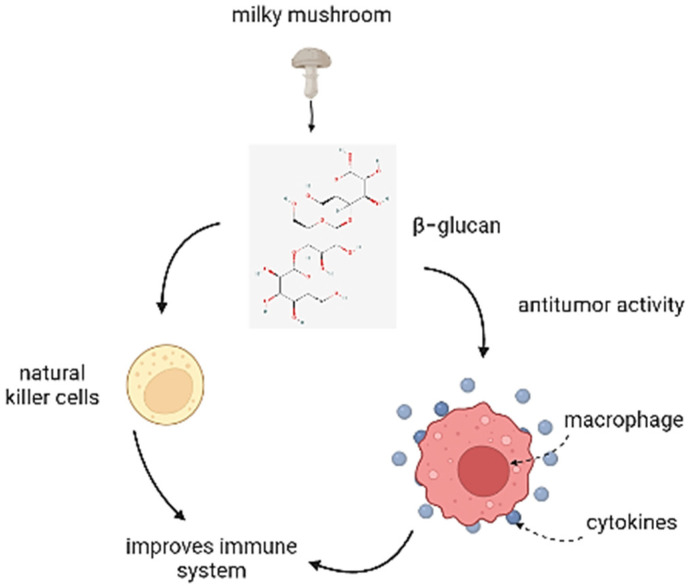
Anti-inflammatory mechanism of *Calocybe indica*.

Although inflammation is a barrier function in the body, it is undesirable in the imbalance of self-tissue and might be the source of serious diseases and injuries [62]. The denaturation of protein is one of the causes of inflammation, and according to the majority of researchers and inflammatory diseases it can be controlled by a methanolic extract of *Calocybe indica*, which inhibits protein denaturation and suppresses autoantigen synthesis [21]. Polyphenols and flavonoids were identified in a methanolic extract of milky mushrooms, which may be responsible for their anti-inflammatory properties. The anti-inflammatory efficacy of the extract of *Calocybe indica* might be attributed to the phytochemical components revealed in the extract by GC-MS analysis, namely ethyl tridecanoate, diallyl divinylsilane, 3-phenyl-pyrrolo (2,3-) pyrazine, and N,’-trimethyl diphenethylamine. According to Das et al. (2021) [22], the β glucan present in milky mushrooms is also responsible for the anti-inflammatory and antitumor activity which enhances the immune system (Figure 5).

## 5. Applications in the Food Processing Industry

The urge for functional foods from natural resources over synthetic ingredients is rising with age in the industry and foods could be available as dietary supplements, pharmafoods, phytochemicals, and myco-chemicals [15]. Mushrooms are used in a variety of ways, including as meals, dietary supplements, and medicines known as “mushroom pharmaceuticals” [63]. Since *Calocybe indica* is a micro-fungus that accumulates nutrients and minerals from the substrate in which it grows, it might be a potential option for production and can be used as a supplemented food source. Enrichment of *Calocybe indica* is a new method that can reduce total manufacturing costs and improve the quality of *Calocybe indica* for consumption and nutraceutical development by employing a variety of renewable and less expensive supplementary substrates [64].

### 5.1. Breakfast Recipes

A mushroom supplementation using powder of *Calocybe indica* was provided to human volunteers by Anju and Ukkuru (2016) [11], and after preliminary screening, the following three separate volunteers were chosen: hyperglycemic, hyperlipidemic, and hypertensive, while eliminating the those who were on medication. Various standardized recipes (breakfast dishes such as dosa, chapati, mixing the powder in curd, black tea, chutney, and others) were used to ensure the prompt incorporation of mushroom supplements in everyday meals, and blood sugar, blood pressure, and cholesterol levels were measured at the end of three months, with diabetic, hypertensive and hypercholesterolemic respondents showing a marked decline to normal levels, respectively.

### 5.2. Bakery Products

Similarly, in a recent study conducted by Rathore et al. (2019) [65], *Calocybe indica* powder was incorporated into cookies and it was found to possess high-quality protein, with high dietary fiber, β-glucan, and excellent antioxidant activities including phenols and flavonoids. Hence, the study concluded that the *Calocybe indica* powder could be used as an emerging ingredient for formulating bakery products comprising improved nutritional and nutraceutical properties. The physicochemical and textural properties of the bakery products changed with the addition of mushroom powder and the volume of cakes also increased with an increase in mushroom powder levels [66].

### 5.3. Other Food Items

*Calocybe indica*, the most studied species, are regularly consumed in soup, stir fry recipes, toppings of pizzas, curries, and many recipes [67]. However, in a study conducted by Shirur et al., 2014 [68] three recipes (Mushroom curry, Pickle Salad, and any other recipe) were prepared with the use of five varieties (White button, Oyster, Paddy straw, Shiitake, and milky mushroom) to analyze the response of the individuals consuming the food. Herein, the milky mushroom was preferred by very few respondents as their first preference.

### 5.4. Cooking Methods

A study conducted by Arora et al. (2014) [69] concluded that since mushrooms are rarely consumed raw, and instead cooked or processed into a variety of dishes, a systematic investigation was conducted to determine the effects of various cooking processes, such as boiling, microwaving, and stir-frying (in sunflower oil), on the antioxidant activity of *Calocybe indica*. The DPPH radical scavenging capability of *Calocybe indica* was found to be greatly reduced during the microwaving and boiling processes; however, stir-frying increased both the activity and phenolic content (970.03 mg gallic acid equivalent/L of extract) and it has been suggested that stir-frying as a cooking method might improve the beneficial characteristics of *Calocybe indica*. It has been concluded that milky mushrooms might be employed as an additional nutrient in food items since it is a novel functional food element for a healthy lifestyle [15].

## 6. Pharmaceutical Approaches

Due to their effective nutritional and therapeutic functionalities, wild mushrooms have attracted a lot of attention in the disciplines of medicine and food processing in recent years [27]. According to various studies, knowledge concerning the health benefits and functioning mechanisms of mushrooms supplementation have developed a captivating interest in the food nutrition area, and for the formulation of a more balanced diet pattern by all human, which will pave a new way for the prevention even cure of some major diseases such as cancer, heart and nervous ailments [70]. It is ideal for individuals with hyperacidity and constipation because of the alkaline ash and high fiber content [5]. Due to the presence of phenolic chemicals, terpenes, polyketides, sterols, ergosterol, flavonoids, and steroids, *Calocybe indica* is used as a dietary supplement as well as a pharmaceutical. According to research, this particular species of mushroom contains p-coumaric, syringic, caffeic acid, and many other polyphenolic compounds [22]. Several research findings have revealed that *Calocybe indica* is effective in lowering total plasma cholesterol and triglyceride levels [37,71]. *Calocybe indica* polyphenols and flavonoids protect against oxidative damage caused by free radicals and reactive oxygen species. As a result, it suppresses the onset of diseases including aging, carcinogenesis, obesity, and diabetes. *Calocybe indica* is also used as an anti-diabetic by indigenous people [72]. In a study conducted by Chatterjee et. al. (2011) [58], the effect of ethanolic extract of *Calocybe indica* was tested against carbon tetrachloride (CCl_4_) induced hepatic damage in mice. The results suggested that ethanolic extract of *Calocybe indica* protects CCl_4_-induced chronic hepatotoxicity in mice by restoring the liver antioxidant status. Different extracts of milky mushrooms have been found to possess versatile bioactivities which justify the theorem of one drug multiple targets, in which ethanolic, oven-dried ethanolic extract and lyophilized ethanolic extract, methanolic extracts, crude polysaccharides, hot aqueous extract, petroleum ether extract, water extract were contributors to versatile beneficial activities [15].

### 6.1. Anti-Obesity

In an investigation, the hypercholesteremic effects of *Calocybe indica* on healthy human subjects demonstrated a significant reduction in the cholesterol levels of the individuals. The participants in this study, on the other hand, had a normal BMI of 20 and had borderline elevated LDL-c values (230 mg/dL) [11]. Increased LDL-c, decreased HDL-c, and increased triglyceride levels are considered to be indicators of obesity [71]. Other edible mushrooms, such as *Agaricus bisporus* and *Hericium erinaceus*, are shown to have anti-obesity properties [73]. In a study of zebrafishes with short-term high-fat diet induction approached by Nagaraj et al., 2021 [57], Atorvastatin treatment was shown to be as effective as 50 μg of the extract. A total of 200 μg of the extract resulted in a drop in BMI levels, bringing them closer to values seen in the control group. Treatment with *Calocybe indica* suppressed body weight and fat distribution in male and female zebrafish. It has been previously reported by Anju et al., 2016 [11], that the hypercholesterolemic effects of *Calocybe indica* are consistent with these results. Proteins with biological activities have also been found, that can be used in biotechnological aspects and the development of new drugs including lignocellulose–degrading enzymes, lectins, protease and protease inhibitors, and ribosomes–inactivating protein hydrophobins.

### 6.2. Antidiabetic Activity of Calocybe indica

**Figure 6:**
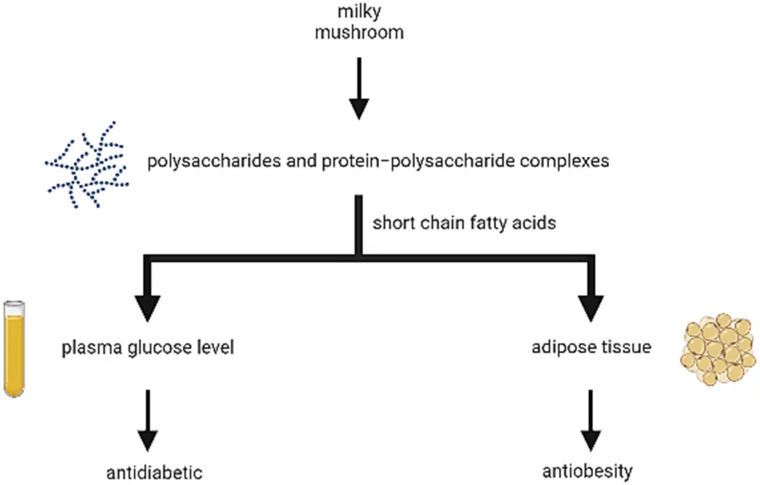
Antidiabetic and anti obesity mechanism of polysaccharides and protein-polysaccharide complexes in *Calocybe indica*.

Glucose levels in the blood and urine are raised in diabetics, leading to excessive urination, thirst, hunger, and complications with fat and protein metabolism. Insufficient insulin secretion or insulin resistance are both causes of hyperglycemia. In diabetics, insulin deficiency impairs glucose utilization, which leads to an increase in oxygen-free radical production. An insulin insufficiency causes a variety of biochemical and physiological modifications. Insulin estimates are considered an indicator of β-cell function. The antidiabetic activity of milky mushrooms in vitro and in vivo was dosage dependent, with substantial findings for α-amylase and α-glucosidase activity. At the studied dose level (200 mg/kg body wt.), the methanolic extract exhibited substantial action, comparable to glibenclamide, a typical anti-diabetic medication [19]. The presence of polysaccharide and protein-polysaccharide complexes in milky mushrooms has also been found to show antidiabetic results (Figure 6) [21].

## 7. Conclusions

It can be concluded that the investigated edible mushroom, *Calocybe indica*, is a good source of food in terms of protein, carbohydrate, fiber, amino acids, energy values, and a good array of vitamins and minerals present. Due to their nutritional values, these mushrooms may provide significant support against malnutrition diseases. Furthermore, in light of the current nutrient deficiency and health problems all over the world, the regular consumption of *Calocybe indica* mushrooms can play an important role in health and disease prevention. Because of its incomparable productivity and shelf life to any other cultivated mushrooms in the world, the milky white mushrooms could play an important role in satisfying the growing market demands for edible mushrooms.

## Figures and Tables

**Table 1:** Suitable methods for the estimation of the nutritional profile of *Calocybe indica*.

Proximate	Composition of Components	Suitable Estimation Methods	References
Protein	Alanine (16.05%) Arginine (2.37%) Aspartic acid (11.85%) Glutamic acid (14.75%) Glycine (7.41%) Histidine (8.07%) Isoleucine (12.37%) Leucine (5.17%) Lysine (2.26%) Methionine (0.27%) Phenylalanine (2.29%) Serine (6.78%) Threonine (3.70%) Tyrosine (3.42%) Valine (4.33%)	Kjeldahl method Lowry method Bradford method	[4,18]
Vitamins	Retinol (0.32 mg/100 g) Vitamin B (Thiamine Riboflavin Pyridoxine Pantothenic acid Nicotinic acid Folic acid Cobalamin) (0.35 mg/g) L-ascorbic acid (1.03 mg/100 g) Calciferol (78.33 µg/g) Tocopherol (2.8 mg/100 g)	High-Performance Liquid chromatography Spectrophotometry Colorimetry Fluorometry	[18,37]
Minerals	Potassium (28209 ppm) Magnesium (1012 ppm) Phosphorous (381 ppm) Barium (9.3 ppm) Iron (77.55 ppm) Aluminium (38.92 ppm) Manganese (20.56 ppm) Copper (28.20 ppm) Zinc (35.12 ppm) Boron (18.87 ppm) Nickel (0.85 ppm) Chromium (0.89 ppm) Selenium (0.0132 ± 0.001 ppm) Arsenic (0.54 ± 0.004 ppm) Calcium (20.65 ± 2.1 ppm)	Atomic absorption spectrophotometry	[4,24,43]
Carbohydrates	(Glucose Galactose Fructose Xylose Mannose Fucose Rhamnose Arabinose Trehalose Mannitol (1→3) β-linked glucose (1→6) β-linked glucose) (50.03 kcal/100 g)	Thin-layer chromatography, Gas chromatography, and High-Performance Liquid chromatography	[7,40,44]
Fatty acids	arachidic acid (0.28%) dichomo-linolenic acid (0.657%) eicosapentaenoic acid (1.86%) elaidic acid (22.47%) erucic acid (0.34%) gondoic acid (1.24%) heneicosylic acid (0.41%) lauric acid (1.42%) lignoceric acid (1.57%) linoleic acid (42.88%) margaric acid (0.27%) myristic acid (1.49%) myristoleic acid (1.56%) palmitic acid (1.30%) palmitoleic acid (0.21%) pentadecyclic acid (0.65%) stearic acid (20.36%)	gas chromatography	[4,45]

**Table 2:** The activity of *Calocybe indica* identified by several extraction methods.

Bioactivity	Compounds	Effects	References
Anti-oxidant	Crude polysaccharide Ergothioneine Glutathione	The antioxidant assays revealed strong potential free radical scavenging potential as well as effective reducing power at the highest concentration (10 mg/mL) tested.	[41]
Anti-cancer	Ethanolic extract Crude polysaccharides Polysaccharide peptide complexes	Strong antiproliferative effects against the tested cell lines within the concentration range of 100–500 µg/mL. The extract impedes cell migration and induces apoptosis through activation of the intrinsic pathway. This was the first report of the anticancer effect of ethanolic extract from *Calocybe indica* on human pancreatic cancer.	[57]
Anti-obesity	Hot aqueous extract Squalene Protein-polysaccharide complexes	Excellent anti-obesity effect in diet-induced obese zebrafish model was observed wherein, treatment with 200 µg extract, a dose-dependent decrease in blood glucose, cholesterol, and triglyceride levels which had increased due to a high-fat calorie-rich diet. Furthermore, less lipid accumulation and decreased lipid droplet size in the treated fishes were observed.	[57]
Hepatoprotective	Ethanolic extract	Oral administration of 150 mg/kg body wt. dosage for one week (once daily) protected the mice from hepatic damage induced by carbon tetrachloride in experimental mice by restoring the elevated serum marker enzyme level. The antioxidant status was also improved to normal after the treatment with the extract.	[58]
Anti-aging	Crude polysaccharide	Orally administered 400 mg/kg body wt. dose significantly increased the levels of antioxidant enzymes. D-galactose induced mice showed elevated levels of malondialdehyde which is reported to be associated with aging but upon treatment, a significant reduction in malondialdehyde content was observed in serum and brain tissues.	[41]
Antimicrobial	ethyl tridecanoate undecanoic acid ethyl ester diallyl divinylsilane 3-phenyl-pyrrolo (2,3-) pyrazine Phytol	Inhibition zone measurement against Escherichia coli and Staphylococcus aureus.	[15,59]
Anti-inflammatory	Catechin Syringic acid p-coumaric acid Caffeic acid ethyl tridecanoate diallyl divinylsilane 3-phenyl-pyrrolo (2,3-) pyrazine N,’-trimethyl diphenethylamine	Inhibition of carrageenan-induced acute inflammation.	[21,59]
